# Infectious Disease Transmission during Organ and Tissue Transplantation

**DOI:** 10.3201/eid1808.120277

**Published:** 2012-08

**Authors:** Melissa A. Greenwald, Matthew J. Kuehnert, Jay A. Fishman

**Affiliations:** Food and Drug Administration, Rockville, Maryland, USA (M.A. Greenwald);; Centers for Disease Control and Prevention, Atlanta, Georgia, USA (M.J. Kuehnert);; Massachusetts General Hospital, Boston, Massachusetts, USA (J.A. Fishman);; and Harvard Medical School, Boston (J.A. Fishman)

**Keywords:** disease transmission, infectious diseases, prevention and control, screening methods, screening trends, organ and tissue transplantation, tissue donors, tissue and organ procurement, viruses, virus diseases, molecular biology, epidemiology, diagnostic tests

## Abstract

Infectious disease transmission through organ and tissue transplantation has been associated with severe complications in recipients. Determination of donor-derived infectious risk associated with organ and tissue transplantation is challenging and limited by availability and performance characteristics of current donor epidemiologic screening (e.g., questionnaire) and laboratory testing tools. Common methods and standards for evaluating potential donors of organs and tissues are needed to facilitate effective data collection for assessing the risk for infectious disease transmission. Research programs can use advanced microbiological technologies to define infectious risks posed by pathogens that are known to be transplant transmissible and provide insights into transmission potential of emerging infectious diseases for which transmission characteristics are unknown. Key research needs are explored. Stakeholder collaboration for surveillance and research infrastructure is required to enhance transplant safety.

## Abstract 2

Transplantation of organs and tissues (bone, tendon, skin, cornea) will always be associated with some risk for transmission of infectious diseases from donor to recipient. Understanding and minimizing this risk is difficult for many reasons: donor screening processes vary, screening for every infectious organism is not possible, and assessment of recipient health after transplantation to determine possibility of disease transmission is often not adequate. In May 2010, the US Food and Drug Administration held a meeting to address these challenges and establish a research agenda for minimizing these transplant transmission risks. Attendees agreed that the focus should be on standardizing donor screening, compiling disease transmissibility data, monitoring of transplant recipients’ health, and assessing effectiveness of measures to minimize disease transmission. Collaboration and sharing of perspectives, experiences, and resources of all stakeholders in the transplantation process (government, private industry, and health care providers) can improve the safety of organ and tissue transplantation.

Multiple clusters of infection associated with allograft transplantation and poor outcomes have been described for recipients. These clusters included infection transmitted to recipients of vascularized organs or tissues such as bone, tendon, skin, or corneas. The exact risk for infection associated with organ or tissue transplantation is unknown but is related to multiple factors, including epidemiology of specific infectious exposures, tissue tropism of the organism, and transmissibility of potential pathogens through transplantation. Even for known pathogens, there are few data on the microbial characteristics that determine transmissibility. Similarly, with regard to new pathogens or pathogens that are found in new regions or populations, i.e., emerging pathogens, there are few data regarding optimal approaches to assessing risks of allograft-associated transmission.

Transplantation of organs and tissues is increasing (Figure). Prospective assessment of the risk for allograft-derived infection is complicated by the variety of potential pathogens and technologies required for detection and by variability between allograft recipients. Such infections must be distinguished from other transplant-associated infections, including nosocomial infections and infections derived from tissue contamination during handling or processing.

**Figure:**
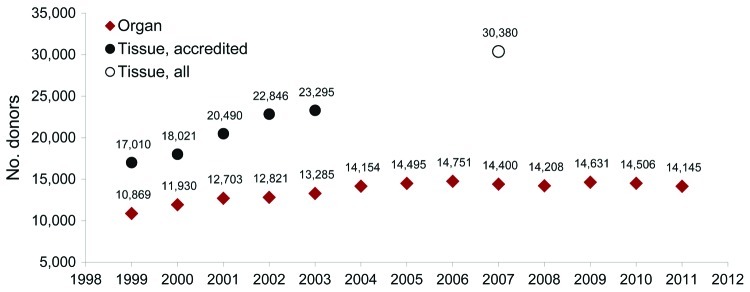
Number of deceased and living organ donors and deceased tissue donors, United States, 1998–2012. Organ donor data source: Organ Procurement and Transplantation Network. Tissue donor data source: American Association of Tissue Banks (AATB) survey data. Survey data for tissue donors includes only AATB-accredited tissue banks, except in 2007, when data were collected from accredited and nonaccredited tissue banks. No information is available regarding the number of organ and tissue donors.

In an attempt to prevent donor-derived infections in transplantation, organ and tissue donors are evaluated to identify those that might be more likely to harbor transmissible pathogens. Current donor evaluation protocols rely on reviewing the potential donor’s epidemiologic and clinical history (i.e., donor screening) and communicable disease test results (i.e., donor testing). Donor screening methods include evaluating the donor’s medical history and physical examination results and assessing (often in the form of a questionnaire) the donor for behavioral risk factors associated with a higher prevalence of communicable diseases. Donor testing includes the donor’s microbial culture data (e.g., blood, urine, sputum), serologic assay results (e.g., antibodies against HIV, hepatitis B virus [HBV]), and hepatitis C virus [HCV]), and increasingly, nucleic acid testing (NAT) results, including assays for HIV, HCV, or HBV. Although regulatory requirements and risk-benefit considerations for evaluating organ and tissue donors differ, the fundamental process for donor screening and testing, and the challenges faced in prospectively assessing the risk for donor-derived infection, are similar for organ and tissue donors. Despite the recognized need to address these challenges, there is little consensus regarding direction for improvements in donor evaluations or for identification of future epidemiologic threats posed by allograft transplantation.

In May 2010, the US Food and Drug Administration held a workshop entitled Emerging Infectious Diseases: Evaluation to Implementation for Transfusion and Transplantation Safety. The goal of this meeting was to identify a research agenda to characterize the risk for transmission of donor-derived infections and inform the development of guidelines for emerging infectious diseases (*1*). The major issues identified are summarized in this review.

## Challenges Identifying Potential Pathogens in Transplantation

Identification of gaps in current knowledge regarding disease transmission by transplantation will facilitate development of a research agenda for this field. Despite recognition of possible donor-derived infections in recipients, when investigating such events, confirmation of an association with the transplanted organ or tissue (i.e., imputability) is often uncertain. The incidence of recognized infectious disease transmission in organ recipients is estimated at ≈1% on the basis of limited data (*2*). Uniquely for tissue transplantation, the incidence of transmission may be further reduced by postprocurement processing. Given the immunocompetence of tissue recipients and routine use of antimicrobial drug prophylaxis, disease transmission appears to be rare. In the face of a recognized recipient infection, it is challenging to determine whether an infection was donor-derived, recipient-derived, nosocomial, or caused by allograft contamination. Policy development regarding optimal donor screening and testing practices will require more complete data on disease risk and extent of transmission.

### Threat Identification

Identification of potential infectious disease threats can be accomplished by systematic approaches, including outbreak investigations and literature searches, and through routine, intensive searches for new pathogens. Donor-derived infectious disease risks posed to recipients by organ and tissue transplantation have been identified primarily through published descriptions of clusters of allograft recipients with infections, sometimes caused by unusual organisms such as lymphocytic choriomeningitis virus (*3**,**4*) or rabies virus (*5*), or after transmission events associated with blood products. Because viable, transplanted organs are highly efficient vectors for microbial transmission into immunosuppressed hosts, transmission events in organ transplant recipients may serve as sentinel events for emerging infections.

### Changing Epidemiology

Identifying potential infectious exposures is accomplished, in part, by obtaining a donor epidemiologic history. Potential organ and tissue donors may reside in, or have migrated between, geographic regions where different organisms are endemic, which confound screening and testing efforts. The changing geographic distribution of pathogens such as West Nile virus (WNV), chikungunya virus, dengue virus, *Babesia* spp., and *Trypanosoma cruzi* (i.e., Chagas disease) have resulted in clusters of infections transmitted to organ recipients in regions where the pathogens are not endemic. Epidemiologic shifts and other disease transmission risks discussed below illustrate the need for systematic risk-based approaches to evaluating the transmissibility of pathogens through tissue and organ transplantation.

### Transmissibility of an Organism by Transplantation

The transmissibility of an organism by transplantation is generally imputed by after-the-fact recognition of the organism in the blood or tissues of the allograft donor and recipient. Detection and reporting of transmission events is incomplete. The biology of disease transmission from allografts has not been well studied, even for organisms known to be transplantation transmissible. More accurate risk assessment requires data regarding the epidemiology and transmission characteristics of a specific organism in a specific graft type.

Transmission of infection is dependent on a series of factors that are organism and host dependent (Table 1). These factors include the organism type (virulence) and the presence or absence of effective host immune and inflammatory responses. Increasingly potent immunosuppressive agents used to prevent rejection in organ transplant recipients have also increased risks for opportunistic infections and viral infection–mediated malignancies, further complicating the determination of whether a posttransplant event is donor derived.

**Table 1:** Factors involved in transmission of infection by human allografts

Factor	Specific feature
Organism	Incidence, virulence, tissue tropism
Host	Immune status, prior immunity, general health
Graft	Organism incidence, viability, receptors for organisms, processing
Medical staff	Clinical experience, laboratory technical support, comprehension of assay characteristics
Epidemiology	Donor exposures (outbreaks, nosocomial, vectors, animals)

### Experience with Allograft-associated Transmission of WNV

The pathogenesis of WNV infection illustrates the complexity of disease detection and prevention in organ transplantation. WNV is asymptomatic in 80% of immunocompetent persons infected by mosquito bites. WNV viremia in blood donors is typically detected within 1–5 days after infection, depending on whether testing is performed by using individual donation or minipool tests. Detecting WNV viremia may be complicated by low-level viremia. WNV viremia in blood donors generally clears within weeks, although viremia may persist despite the appearance of antibodies within 7–10 days after exposure (*6*). The value of reports of persistent detection of WNV nucleic acid in urine of some persons years after infection remains to be determined (*7*).

WNV antibodies do not always protect susceptible cells from infection in vitro (*6*). In general, the likelihood of central nervous system involvement with WNV infection is greater in immunosuppressed hosts than in healthy persons (*8*). In all reported organ donor–derived infections with WNV in the United States, 2 of 4 kidney recipients showed development of neuroinvasive disease (WNND) but recovered, 1 showed development of virema and seroconverted but remained asymptomatic, and 1 did not demonstrate transmission. In 2 liver transplantation recipients, 1 showed development of WNV fever but recovered, and another showed development of WNND and permanent neurologic injury. Two heart recipients showed development of WNND but recovered. A recipient showed development of WNND but never recovered (*9**–**11*). Variability in transmission patterns among organ recipients exposed to WNV illustrates the need for studies that will define the organism and host factors governing transmission. Such data also will provide a basis for studies of emerging infectious diseases with unknown transmissibility characteristics.

Donor-derived disease transmission reports from tissue transplantation are relatively infrequent. WNV also illustrates some of the challenges faced in detecting donor-derived transmission events in tissue recipients. In contrast to organ- and blood-derived infections, tissue transmission of WNV has not been reported. Lack of similar reports of WNV transmission to tissue recipients may reflect underrecognition, i.e., differences in transmissibility, clinical symptoms, diagnostic approaches, immunosuppression, clinical follow-up of the recipient, and underreporting (*12*). Detection of infections after tissue transplantation might be delayed when a donor-derived infectious source is less likely to be considered. Clinicians often consider tissue allografts safe despite published reports (*13**–**19*). Therefore, disease detection is dependent on skills, knowledge, and heightened awareness of clinicians caring for allograft recipients. Despite the absence of reported WNV transmission events, the large volume of tissue grafts and the potential availability of >100 tissue products from individual donors indicate the need for assessing and optimizing methods for identifying potential pathogens in tissue transplantation.

## Challenges in Evaluating Donors

There are knowledge gaps in the efficacy of current practices in evaluating, i.e., screening and testing, donors for infectious agents. Screening identifies donor risk factors, but the sensitivity and specificity of current approaches are largely unknown. Serologic assays detect chronic or persistent infections but are less useful for diagnosing more recent infections. Some cases of donor-derived infection in organ transplantation occurred after failures in serologic testing (e.g., window-period cases before seroconversion) (*9**,**19**–**21*). NAT is useful for detecting infection only in blood samples of viremic donors and is not available for every potential organism (*22*).

There is variability in the performance (between specific tests and laboratories) and application (selection of a particular assay by the program performing the test) of assays used in donor testing. This variability limits the ability to compare and interpret existing testing data derived from donor populations that could, in turn, inform decisions regarding optimal assay selection. For example, some organ-procurement organizations use assays indicated for donor testing and others use diagnostic tests. Programs use antibody assays from different manufacturers (resulting in differing performance characteristics), and some use NAT routinely or only in special circumstances (e.g., on the basis of donor characteristics). There are few data regarding the clinical performance of these assays in donor populations.

## Additional Challenges

### Posttransplant Surveillance

In the absence of routine active surveillance (e.g., posttransplant testing of all recipients to ascertain transmission events), donor-derived infectious disease transmission remains difficult to recognize and document. Current surveillance systems that identify transmission events are passive (i.e., clinicians must diagnose infection, recognize a relationship with transplantation, and report the event). Given the asymptomatic nature of some acute infections with serious, but delayed, sequelae (e.g., HBV), it is unlikely that most transmission events could be detected by passive surveillance alone. Active surveillance would increase knowledge of transmissibility and might detect emerging infectious agents within recipients of organs or tissues. Transmission rates are likely to vary among donor and recipient groups (e.g., by epidemiologic exposures) and among organs and various tissues.

### Posttransplant Reporting

There are multiple, potentially confusing, pathways for adverse event reporting (Table 2). The Joint Commission in the United States requires accredited centers to report to manufacturers (i.e., establishments that process and distribute human tissues for transplantation) any transmissions possibly associated with human tissues. Manufacturers must investigate such reports and report to the Food and Drug Administration serious adverse reactions for which there was a reasonable possibility that the cell or tissue caused the reaction (*23**,**24*). Such reporting depends on clinician recognition and reporting of an allograft-derived infection; underreporting is a recognized limitation of such programs. Given the general lack of awareness of the potential for donor-derived infection and multiple common sources of infection in a transplant recipient, such as the surgical procedure or nosocomial infection, there is great potential for underreporting. Potential organ-associated disease transmissions (infection and malignancy) are reported to the Organ Procurement and Transplantation Network (OPTN). In addition, state and local health departments may also require notification for transplant transmissions. For all of these reporting systems, limitations include lack of uniform reporting criteria and coordinated data collection.

**Table 2:** Organizations involved in detection, investigation, and communication of organ and tissue transplant–associated infections

Organization and website	Role
Food and Drug Administration	Regulates transplantation of human cells and tissues and requires Food and Drug Administration–regulated tissue establishments to investigate and report serious adverse events related to infectious disease transmission
Organ Procurement and Transplant Network	Operated by the United Network for Organ Sharing under contract with the Health Resources and Services Administration
Reporting Line	Reporting mechanism for potential patient safety issues and Organ Procurement and Transplant Network policy violations
Disease Transmission Advisory Committee	Organ Procurement and Transplant Network committee that compiles all potential transplantation-transmitted infection cases reported to the United Network for Organ Sharing
Council of State and Territorial Epidemiologists	Public health reporting and investigation of suspected transplant–transmitted notifiable diseases and illness clusters are coordinated by public health epidemiologists, as described in a 2010 position statement
Centers for Disease Control and Prevention	Provides assistance to health departments on organ and tissue transplant–associated adverse events
The Joint Commission	Accredits and certifies >18,000 health care organizations and programs in the United States; mandates reporting of tissue adverse patient reactions to the donor source facility

### Coordinating and Sharing Aggregated Donor Screening and Testing Data

One deceased donor may supply organs, eyes, and numerous other tissues, which may be widely distributed over time and geographically. Some deceased donors are tested many times (by organ procurement organizations (OPOs), eye banks, and tissue banks). Collecting and sharing of donor data are challenging in the absence of linked, unique identifying information for each donor (e.g., donor identification number) (*25*) and centralized data sharing systems. Epidemiologic data (e.g., incidence of infections in the donor population by allograft type and geographic region) and other data (e.g., donor test results correlated with individual recipient testing) need to be aggregated and accessible by contributing OPOs, eye banks, and tissue banks to assess the associated transmission risk. These data could be compared with information collected from blood screening tests for the same donors. Efforts were initiated to improve communication of recipient outcome data between the organ and tissue transplant communities in a pilot project. However, major issues remain to be addressed, such as standard definitions and incentives for participation, before a useful system could be deployed nationally (*12*). Such data could be used in evaluating effectiveness of current donor screening and testing strategies and developing standardized research methods for use in assessing donor evaluation tools.

### Quality of Donor Screening and Testing Data

Donor screening protocols should reduce the likelihood that tissues or organs that would transmit infection will be procured, while preserving availability (avoiding false-positive test results). Effectiveness of current donor screening procedures has not been systematically evaluated. To develop data useful for assessing donor screening practices, high-quality data must be collected by using standardized protocols and assays of known performance characteristics. The nonuniformity of protocols used to screen donors among various organizations impedes critical assessment of donor evaluation protocols. One initiative to bridge this gap is the development and validation of the proposed Uniform Donor History Questionnaire for Organ, Tissue, and Eye Donors (*26*).

Screening donors through donor history questionnaires can reduce transmission risk only to the degree of the accuracy and completeness of the information provided. This effort is limited by obtaining information by proxy (i.e., from the families of deceased donors) and incomplete information about prior infectious exposures. As a result, testing donor specimens for known transmissible organisms is required to further reduce risks by excluding the infected potential donor or guiding clinical care of recipients. Given the changing epidemiology of infection, a major challenge is developing new assays for emerging infectious diseases. New assay development is typically resource-intensive and too slow to reflect rapid shifts posed by epidemic disease (e.g., assays for severe acute respiratory syndrome), but can be accomplished over time (e.g., assay development during a WNV infection outbreak). New approaches to assay development and for evaluating such assays in organ and tissue donor populations, together with streamlined approaches to evaluating assay effectiveness in a manner appropriate to regulatory review, could enable more effective responses to threats of emerging infectious diseases.

## Addressing Gaps: Key Research Needs

There are immediate clinical needs (e.g., optimizing assays and information sharing between suppliers and clinical centers) and long-term research opportunities in the field of allograft transplantation. A structured approach to addressing gaps in scientific knowledge, perhaps through an overarching Department of Health and Human Services strategy, is needed to enhance the safety of organ and tissue transplantation. The data required fall into several general areas as described below and summarized in Table 3.

**Table 3:** Key research factors and needs for organ and tissue transplant–associated infections

Factor	Need
Denominator data	Tissue transplantation: number and type of allografts transplanted
	Discard rate: number of donors rejected for organ and tissue transplantation because of identified donor risk
Transmissibility data	Preclinical: animal studies, studies of unused organs, or tissues from infected donors
	Clinical studies: outcomes from increased risk organ donors
Risk mitigation strategies	Processing: tissue processing, pump perfusion of organs
	Effects of antimicrobial agents: in organ donors, in organ or tissue recipients
Donor questionnaire	Effectiveness of questions for obtaining accurate answers and for identifying donors with higher likelihood of having a positive test result
Research infrastructure	Organized network: to collect, share, and analyze data in organ and tissue transplantation
	Harmonization: product names, label information, data collection methods, data sharing mechanisms
Risk assessment	Use of data: model risks, further refine research needs
	Quality improvement: evaluate effectiveness of changes, refine models for use with emerging diseases

### Denominator Data

Baseline data for the number and types of tissue grafts distributed each year are available only through voluntary reporting to the American Association of Tissue Banks and are limited by a lack of coding and traceability after tissues are delivered to end users (e.g., hospitals, dental clinics, surgical centers). Individual tissue banks rely on hospitals to return implant cards to assess use of allograft materials; compliance is voluntary and accuracy of the data is limited by incomplete return rates. Regulatory mandates require OPO and organ transplant programs to report extensive data to the OPTN on all solid-organ donors (living and deceased), candidates on the waiting lists, and recipients. However, the discard rate of donor organs on the basis of microbiologic testing are not collected by OPTN and are retained at the local OPO rather than the national level. Improved testing might change the denominator by increasing or decreasing the number of available allografts for transplantation; collection of these data is needed to guide decisions regarding optimal donor testing in organ transplantation (*27**,**28*).

### Seroprevalence Data

The prevalence of infectious disease in potential organ and tissue donors has not been systematically evaluated. Studies of blood and organ donors suggest that the probability of viremia for HIV, HCV, HBV, and human T-cell lymphotropic virus in the United States is higher in tissue and organ donors than in first-time blood donors (*29**,**30*). Prospective data collection is needed to define baseline seroprevalence in different donor populations; these data could be used to develop enhanced strategies for donor screening and testing to prevent disease transmission.

### Transmissibility Data

Data are needed regarding the transmissibility of potential pathogens by the type of organ or tissue transplanted. Current programs focus on excluding donors with risk factors for, or serologic markers of, blood-borne pathogens. The transmissibility of infection from organs or tissue donors with various nonspecific clinical syndromes (e.g., pneumonia, meningoencephalitis, sepsis) is unknown. Clinical data (based on cultures and serologic studies) and preclinical data from animal studies would enable more evidence-based decisions regarding donor eligibility.

### Risk Mitigation Strategies

Processing methods vary with regard to diverse technologies and strategies used and types of tissue processed. There are few data regarding the effect of tissue processing on decreasing or eliminating infectious organisms. Similarly, the effect on the risk for transmission of infection of antimicrobial drug therapy in donors or recipients is unknown. Increasingly, some organs (notably kidneys) undergo pump perfusion before implantation, but the effect of this manipulation on disease transmission is unknown.

### Donor Questionnaires

As discussed, standardizing and validating current donor screening tools is an essential first step in collecting and analyzing data for donor risk factors and in developing refined strategies for screening and testing donors. Prospective studies comparing results of donor history questionnaires and those of microbiological testing should be performed to evaluate the effectiveness of donor-screening strategies. The availability of tissues and organs for transplantation could be increased without compromising safety.

### Research Infrastructure

There is currently no established network or infrastructure for systematically collecting and analyzing organ and tissue donor data. Given the need for additional data to provide a scientific basis for evaluating and managing allograft donors and recipients, stakeholders must develop mechanisms to prioritize and implement joint research programs. To perform the necessary research, the allograft transplant communities need to develop systems to harmonize labeling nomenclature and data elements (product names and data that are contained on product labels), data collection methods, and mechanisms for data sharing. Elements needed to develop such a research infrastructure will include shared data repositories and software for collecting and analyzing surveillance data from donors and recipients; common epidemiologic data elements; and universal donor identifiers that link all allograft types to the original donor (*25*), protocols, and standardized nomenclature. Such a networked infrastructure is essential for rapid traceability of tissues and organs from common donors when donor-derived disease outbreaks occur in transplant recipients (*12*).

### Risk Assessment

Newly developed datasets derived from these proposed research activities will enable development of risk assessment models similar to those used to characterize or predict infectious disease risks in blood donor populations (*31*). Use of such models would further define targets for future research efforts and for clinical investigations of future outbreaks.

## Conclusions

Disease transmission through organ and tissue transplantation has been documented. Recognizing emerging infectious diseases in organ and tissue transplantation is challenging because of nonstandardization of donor evaluations and data collection, pathogen characteristics, and recipient surveillance. Quantifying risk is further complicated by the absence of data regarding the factors affecting disease transmission. Gaps in systematic identification and characterization of the scope and magnitude of donor-derived infectious disease transmissions through organ and tissue transplantation remain a major hurdle to improvements in assessing risk and in developing more effective donor screening and testing strategies. These gaps can be addressed by a shared, overarching research agenda among the allograft communities. Areas of focus for research include compiling donor evaluation (donor screening and testing) and posttransplant recipient surveillance data and disease transmissibility data (basic mechanisms and clinical factors) and assessing the efficacy of mitigation strategies. Prioritizing the research agenda can be best driven by collaboration between government (i.e., regulatory, public health, policy), industry (e.g., tissue manufacturing, supply, test manufacturers), and the allograft transplant provider community (clinicians, hospitals, professional organizations, OPOs, tissue banks and processors). Each stakeholder has unique perspectives, experiences, and resources to share in enhancing the safety of organ and tissue transplantation and benefit the greatest number of recipients.
